# A Descriptive Review of the Healthcare System and the Provision of Oral Healthcare in the Republic of Sudan

**DOI:** 10.1016/j.identj.2024.05.012

**Published:** 2024-07-20

**Authors:** Demetrio Lamloum, Guglielmo Campus

**Affiliations:** a Department of Restorative, Pediatric and Preventive Dentistry, University of Bern, Bern, Switzerland; b Department of Public Health, Experimental and Forensic Medicine University of Pavia, Pavia, Italy; c Department of Surgery, Microsurgery and Medicine Sciences, School of Dentistry, University of Sassari, Sassari, Italy; d Department of Cariology, Saveetha Dental College and Hospitals, Chennai, India

**Keywords:** Oral health, Healthcare system, Dental workforce, Review, Public health insurance, Sudan

## Abstract

Oral health constitutes a significant public health concern in Sub-Saharan Africa. The precise burden of oral diseases and the adequacy of oral healthcare provision remain often unknown. The present study aims to evaluate key components of the healthcare system in Sudan and the delivery of oral healthcare across eight dimensions: Healthcare System Framework, Financing, Service Delivery, Epidemiology, Prevention, Personnel, Dental Education and Training System, and Health Benefit Package. The evaluation of Sudan's healthcare system and oral healthcare provision utilizing the extended World Health Organization building blocks healthcare systems analysis framework. The evolving healthcare landscape in Sudan is characterized by a transition towards a Bismarckian model, a shift facilitated by the implementation of a National Health Insurance Fund. In 2018, Sudan's total healthcare expenditure surged to 2.4 billion US dollars and dental care accounted for less than 1% of this financial allocation. During the period spanning from 2016 to 2019, there was an expansion in the healthcare infrastructure and utilization rates. The number of healthcare facilities and hospital admissions respectively increased from 2,083 to 3,578 and from 14,967,113 to 22,455,772, while the registered number of dentists in 2019 reached 8,964. Within the framework of the national healthcare system, medical consultations and emergency treatments are encompassed in the oral health benefit package. Sudan's healthcare system grapples with endemic vulnerabilities compounded by recurrent political and economic challenges. Nevertheless, strides towards an insurance-based healthcare system and the upward trend observed in oral healthcare provision and infrastructure assets offer promising prospects for future generations.

## Introduction

Notwithstanding the strides made in management, prevention, and healthcare accessibility, oral diseases persist as a predominant global health concern. Approximately 3.5 billion individuals worldwide suffer from oral diseases, constituting nearly half of the global populace,^1^ alimenting disproportionately affect marginalized and susceptible demographics. In Sudan, the healthcare landscape has encountered considerable disruptions due to endemic national socioeconomic vulnerabilities, political unrest, and civil strife.^2^ Hindrances and disparities impede the health system advancement, resulting in fragmented and underdeveloped oral healthcare provision.

Positioned within the Middle East North Africa region, Sudan is the third-largest African country. It has a populace of 45.6 million individuals and contends with challenges encompassing a predominantly rural population and a dynamically evolving demographic characterized by an escalating burden of Non-Communicable Diseases (NCDs).^2^, ^3^, ^4^, ^5^, ^6^ Following the National Health Sector Policy (2021-2024), the country is aspiring to transition into a welfare state and bolster the national healthcare system through the adoption of Universal Health Coverage (UHC).^7^

Since the removal of former President Omar al-Bashir in April 2019, Sudan has undergone a tumultuous political transition marked by recurrent interim administrations and acute military conflicts leading to significant casualties and widespread displacement of civilians. Since mid-April 2023, conflict between the Sudanese Armed Forces and the Rapid Support Forces has resulted in an estimated 5.4 million internally displaced individuals.^8^ Furthermore, the nation faced outbreaks of cholera/acute watery diarrhoea, dengue fever, measles, and malaria, coupled with assaults on medical facilities, shortages of medical supplies, and pervasive insecurity.^8^ Economic, social, and structural impediments have hindered the implementation of effective disease control measures and investments in healthcare infrastructure, including oral health services.^9^

In Africa, oral diseases afflict over 480 million individuals, with factors such as limited exposure to preventive measures and the increased consumption of sugary beverages and foods exacerbating their prevalence.^10^, ^11^, ^12^ Despite the intertwined nature of prevalent NCDs and oral diseases, Sudanese healthcare maintains a division between oral healthcare and general medical care.^13^, ^14^, ^15^ Oral health remains a marginal problem in most of Africa, resulting in inadequate financial and technical investments that undermine prevention, treatment, and health promotion efforts.^16^ In particular, the oral health workforce in sub-Saharan Africa is lacking, with an average ratio of 3.3 dentists per 100,000 people, significantly lower than the global average, aggravated by the ramifications of the COVID-19 pandemic.^16^ In response, the World Health Organization's (WHO) regional office for Africa has prioritized enhancing oral health within the broader NCDs agenda,^17^ aligning with the global endeavour for UHC by 2030 to foster political commitments towards oral health.^18^^,^^19^

## Aim

The objective of this study is to provide an in-depth examination of Sudan's oral health system, expanding the WHO health systems framework.^20^, ^21^, ^22^, ^23^ This model incorporates the WHO's building blocks alongside epidemiological data tailored to the nation, structured into eight dimensions: Healthcare System Framework, Financing, Service Delivery, Epidemiology, Prevention, Personnel, Dental Education and Training System, and Health Benefit Package.

## Methodology

For each dimension, insights and recommendations were gathered from eminent national experts and key stakeholders in the national oral health sector. From June to December 2021, in collaboration with the Italian Ministry of Foreign Affairs Agency for International Cooperation—AICS (*Agenzia Italiana per la Cooperazione allo Sviluppo*), qualitative unstructured interviews were conducted with key entities, including the Curative Medicine Department of the Federal Ministry of Health, the Oral Health Directorate of Khartoum and Red Sea state, the National Health Insurance Fund (NHIF), and Khartoum University. The interview format included a series of pre-planned questions. The list of guiding topics was supplemented by follow-up and in-depth questions that depended on the interviewee's response. These interviews provided critical information on oral health governance, policies, dental service delivery, staff distribution, health information systems, and dental education.

## Results

### Healthcare system framework

The Sudanese healthcare system, as outlined in the 2006 Constitution, the Local Governance Act of 2003, and the Public Health Law of 2008, places significant emphasis on decentralization of services.^24^ Similarly to neighbouring countries,^25^^,^^26^ decentralization from federal state to the districts endeavours to grant authority to local entities over finance, human resources, organization of services, access to care, and governance. Since the 1990s, the Sudanese health system has shifted from a Beveridge model to an insurance based Bismarckian system.^27^ The 1994 Health Insurance Law introduced the NHIF to allow access to basic health services through a national network of facilities and pharmacies. By 2021, about 81.7% of the population was covered.^28^ The insurance system operates on a contributory basis. Individual subscriptions are determined by monthly income levels. Employees along with their families are entitled to essential healthcare services irrespective of size or existing service costs. The employee contributes 4% of the basic monthly salary, as opposed to the 6% covered by the employer (Supplementary eTable1). The state bears all treatment costs for employees and 75% of prescribed medication expenses, as well as expenses for major and minor operations.^29^ Social insurance coverage has progressively expanded to include voluntary and involuntary members, with compulsory enrolment for the population. Approximately 71% of subscribers represent impoverished and vulnerable groups, typically encompassing entire households comprising family heads, non-working parents, and children (spouses, unmarried daughters, and male offspring up to 18 years old). Students are covered up to the age of 25.

As delineated by Dr. Wael Ahmed Fakihammed, Director of the General Directorate of Health Services at NHIF, policies and plans are devised at three administrative levels: federal, state, and district (also referred to as locality). The overall framework operates under the Federal Ministry of Health and 18 state Ministries of Health. The federal level is responsible for national health policies, plans, strategies, monitoring and evaluation, coordination, training, and external relations. State-level responsibilities involve formulating strategies based on federal guidelines and funding.^30^^,^^31^ Districts focus primarily on implementation and service delivery. Primary Health Care (PHC) is the cornerstone of the system, administered by the Ministry of Health (MoH), with limited exceptions for international cooperation and private sector involvement at federal and state levels. Although over 70% of healthcare expenditure being allocated to hospitals, PHC receives less than 6%. Of the hospital funding, 37% is allocated to salaries and 28% to medications. Despite variations in service levels, the overall funding remains constrained.^29^^,^^30^ PHC coverage extends to 93% of the population, albeit with significant disparities between states and between urban and rural areas. Urban regions exhibit 25% more outpatient services compared to rural areas. Healthcare utilization among the insured population is 1.5 times higher than among the uninsured.^29^^,^^30^ At the end of 2016, only 60% of PHC facilities provided the minimum PHC package, and only one-third were fully operational. Despite investments in training and education, the staff turnover rate remains high.

### Financing

In 2018, Current Health Expenditure (CHE) exceeded 2.4 billion US dollars, equating to 4.8% of GDP or 58.84 US dollars *per capita*. General Government Health Expenditure relies on federal and state government allocations, which contribute 6.6% and 7.8% of CHE, respectively. Private health expenditure constitutes 69.3% of CHE, with nearly 69.2% being Out-Of-Pocket (OOP), or 40.7 US dollars *per capita*. Public sector funds are 24.1% of total healthcare revenues, while the private sector accounts for 69.3%. International assistance contributes 6.6% to healthcare financing. The NHIF contributes 28%. Hospitals and ambulatory care providers absorb most of the funds (89%), with about 46% and 43%, respectively. Health system administration and financing, which include government administration and social health insurance, consume around 2%, whereas preventive care providers utilize 6%. Bilateral organizations and international NGOs spend 2% of funds on direct services.^30^^,^^31^ The 88.8% of CHE is allocated to curative services, covering consultation fees, laboratory and imaging investigations, pharmaceuticals, and other medical consumables. Preventive and public health programs receive approximately 5.9% of expenditure, while healthcare governance and financing administration account for 1.9%. Over 36% of OOP is expended at primary health care centres, primarily for user fees and medications, with 40.8% at general hospitals. Physician's private clinics and specialized hospitals receive 16% and 0.8% of OOP expenditure, respectively. Expenditure on dental care comprises just over 1% (see Table 1). Households allocate 99.5% of health expenses to curative care, with preventive care receiving less than 0.5%. Outpatient curative care services account for nearly 92% of total OOP spending, while inpatient curative care consumes 5.4%.^30^^,^^31^

**Table 1:** Healthcare provision 2018. Federal Ministry of Health.

Providers of ambulatory health services	Amount SDG	Percent	Amount $US
Offices of medical specialists	7,000 million	27.3%	294 million
Dental practices	8 million	1%	329,987
PHC health centres	14,173 million	55.1%	596 million
Other medical practices	3,948 million	15.4%	166 million
Providers of ancillary services	580 million	2.6%	24 million
**Total providers of ambulatory care**	25,709 million	100%	1,080 million

### Service delivery

Since 2016, there has been a consistent increase in both the number of MoH facilities accredited with the NHIF and the overall number of attendees. Dr. Ashwag Abdulrahim, Head of the International Cooperation Directorate at NHIF's Department of Project Development, reported a rise in accredited facilities from 2,083 to 3,578, along with an increase in annual attendees from 14,967,113 to 22,455,772. Notably, the number of benefits provided in the first quarter of 2021 equalled the total provided in the entirety of 2020.^28^ Khartoum state hosts over 45% of the total MoH facilities.^28^ NHIF reimbursements to MoH facilities are structured on a monthly instalment basis, with funds allocated based on claims amounts. At the facility level, reimbursements are divided according to predefined percentages: 40% for health professionals, 40% for equipment and utilities, and 20% to the state.^28^

### Epidemiology

Systematic surveillance of dental health status in Sudan, encompassing both paediatric and adult populations, is deficient, and a comprehensive nationwide epidemiological investigation into oral health has yet to be conducted. Most available data concerning the prevalence of dental caries in Sudan is derived from Khartoum state. One out of every two 12-year-old schoolchildren in Sudan reports experiencing an impact on their daily activities due to caries.^32^^,^^33^ Early childhood caries is prevalent among preschool-aged children aged 3 to 5, with approximately a caries prevalence of 52.4% and a mean decayed, missing, and filled teeth (dmft) index of 2.3.^34^ Prevalence of periodontal disease, as bleeding on probing and a pocket depth of at least 4 mm and clinical attachment loss of ≥6 mm, was observed to be 24%, particularly among specific population sub-groups.^35^ In 2017, the Sudan National Cancer Registry documented 920 cases of oral cancer, constituting 9% of the annual cancer incidence rate.^36^ From 1970 to 1985, 12.6% of the 14,922 cancer cases registered at the Sudan Cancer Registry were attributed to oral cavity cancers.^37^ In Khartoum state, oral cancer ranked as the second most common cancer in both genders, following breast and prostate cancers, comprising 9.4% of all cancers and associated with low survival rates.^38^^,^^39^ According to the World Health Organization (WHO), among 27,382 cancers recorded in 2020, 564 were linked to the lip and oral cavity.^40^ In HIV-infected patients the prevalence of oral manifestations of HIV and AIDS is estimated to be between 50% and 60%. A survey conducted in Omdurman indicated that approximately 76.7% of HIV patients exhibited oral lesions.^41^ Noma (*cancrum oris*) is recognized as a neglected disease of poverty and remains a notable public health concern in certain impoverished African nations, although its occurrence is considered infrequent in Sudan.

### Prevention

In Sudan, the utilization of the *Salvadora persica* plant, commonly referred to as the toothbrush tree, is widespread. *Salvadora persica* has been documented to exhibit antibacterial, antifungal, anticariogenic, and antiplaque properties.^42^ Traditional oral hygiene practices, including the use of chewing sticks (known as miswak or siwāk), crafted from the twigs, stems, or roots of various plant species, persistently advocated for their accessibility, affordability, and simplicity.^43^^,^^44^ Despite the enduring popularity of traditional miswak use, the adoption of toothbrushes and toothpaste is also prevalent.

### Personnel

According to data from the WHO Global Health Observatory, the density of dentistry personnel in Sudan exhibited an improvement from 0.24 per 10,000 population in 2004 to 2.1 in 2015, a notable increase albeit still lower compared to the approximate ratio of 1:2,000 observed in most Western countries.^45^ Dr. Saafran Mohamed Ahmed Alzaki, Director General of the MoH in the Red Sea state, reported that as of 2019, 8,964, is the total number of registered dentists, 519 being specialists. Out of said category, 532 were employed within the MoH, resulting in a density of 0.12 dentists per 10,000 population. Personnel engaged in private practice is not formally registered. The distribution of dentistry personnel appears to be disorganized and concentrated in Khartoum (Supplementary eTable2). Dental laboratory technicians and dental assistants are scarce. The recording system for allied health professionals is deficient. Oral hygienists and dental assistants are legally authorised to practice under the current legislation. Sudan has experienced a brain drain, with approximately 60% of health professionals leaving the country since the early 2000s.^46^^,^^47^

### Dental education and training system

**Figure 1:**
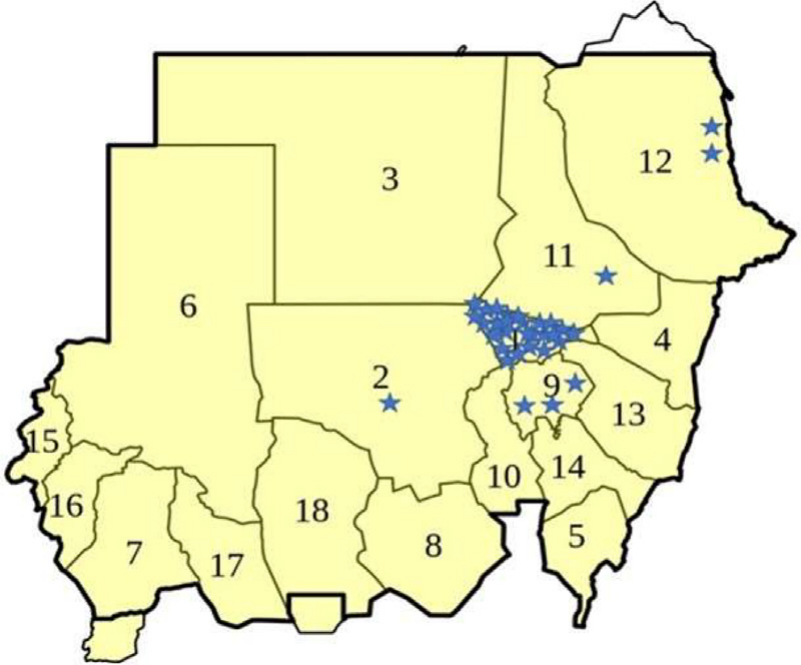
Dental universities distribution. University of Khartoum.

Sudan boasts a rich history of medical education spanning over a century, with institutions such as Gezira University, Khartoum University, and the Sudan Medical Specialization Board.^48^ As for data provided by Dr. Shaza K. Abass, Dean of the Faculty of Dentistry, University of Khartoum, Universities are predominantly concentrated in Khartoum (see Figure 1). Training and the production of healthcare professionals have seen consistent growth, with both public (55%) and private (45%) universities (Supplementary eTable3) experiencing an upsurge.^49^^,^^50^ Gender distribution is largely equal, with a demographic skewed towards young professionals.

The Ministry of Education (MoE) oversees pre-service training and production of health workers across public universities with medical and health science facilities. The Medical Council is responsible for the registration and licensing of doctors, pharmacists, and dentists. Civil service under the Federal Ministry of Health is the primary employer, along with other sectors such as the military, police, universities, and the Health Insurance Fund. Universities offering dental programs in Sudan are situated in major cities, with 33 universities offering such programs, nine of which were under approval in 2021. Upon graduation, dental university students are required to undergo a probationary period in an MoH hospital, with temporary registration provided by the Medical Council. Permanent registration is granted upon completion of the internship. While a system of continuous medical education through seminars and workshops has been established, it lacks formalization, and there is currently no monitoring system in place. Postgraduate programs are offered by both the MoE and the MoH through the Sudan Medical Specialization Board (SMSB). The SMSB offers Medical Doctorate programs, while the MoE offers postgraduate programs, with both institutions also providing opportunities for PhD programs.

### Health benefit package

The general health benefit package includes an extensive group of service: (1) medical consultations; (2) laboratory investigations (routine, chemical, serology, haematology, etc); (3) diagnostic investigations like radiology and imaging diagnostic services (X- rays, U/S, MRI, CT, etc.); (4) surgical operations such general surgery, ENT, GIT, orthopaedics, ophthalmology, urology, obstetric and gynaecological, cardiovascular, dermatology, etc.); other services such as physiotherapy, dental services, and respiratory tract, hearing, cardiovascular, nephrology, psychiatric, and eye care. The dental health benefit package is described in Table 2.

**Table 2:** Dental health benefit package. National Health Insurance Fund.

Dental services included	Dental services exempted
Medical consultation: dentist, general practitioner, and medical assistants	Root canal treatments
Treatment of infection	Fixed prosthetics
Scaling	Removable prosthetics
Diagnostic services (X Ray, CT scan, etc.)	Orthodontics
Extraction	Implanting
Surgical procedures	
Filling	

## Discussion

In light of the findings presented, the Sudanese healthcare system has undergone significant transformations over the past two decades. Political fragility and severe economic challenges continue to impact the stability of national services, including healthcare. Aligning with the current state of the aforementioned system, shifting focus towards UHC represents a progressive step and a potential remedy to address supply deficiencies and healthcare demands. The outlined objectives for oral health within the context of UHC include integrating essential oral health services into the basic health benefit package, aligning the oral health workforce with population health needs and social determinants of health, and ensuring financial protection and inclusion of dental care coverage in health insurance packages, alongside expanding fiscal resources for oral healthcare.^51^

Although the relatively low burden of NCDs in Sudan compared to high-income countries, global and oral health needs are escalating. The increasing demand reveal limitations in healthcare services and highlights accessibility issues, particularly for individuals in the lowest income quartiles, whose access to care is often determined by financial means. The development of preventive measures and supporting community-based programs constitute a viable approach to partially alleviate pressing requirements within the healthcare system. Particularly when customized to target specific demographic cohorts such as children and implemented through school-based initiatives. Such strategies yield compounding advantages throughout an individual's lifespan.^52^, ^53^, ^54^

The predominant focus of care under NHIF revolves around curative therapies (see Table 2), reflecting a therapeutic-cantered model akin to "westernized dentistry."^55^ However, the density of dentists per population is low, scatter, and predominantly concentrated in urban areas. Despite an overall increase in the number of oral health professionals, there is a shortage of cost-effective providers.^56^ The WHO Oral Health Strategy 2023-2030 suggests innovative approaches to oral healthcare workforce planning.^57^ As a low-income country, Sudan can benefit from a team-approach focused on mid-level providers (*e.g.*, dental therapists, hygienists, dental nurses, schoolteachers, and social workers).^58^ Costs for dentist training are often prohibitively high and advanced education should reflect local demand to enhance competencies in public health, health promotion, and disease prevention. Allied oral health professionals are equipped with clinical and public health competencies aimed to prevent and treat prevalent oral diseases through essential oral healthcare and rehabilitation interventions within a primary care framework. This approach prioritizes comprehensive care over reliance on advanced technologies and excessive specialization, which are often dominant practices in such field. Also, the phenomenon of medical brain drain exacerbates the shortage of healthcare professionals, with desirable working conditions, improved education access, political stability, higher salaries, and training opportunities serving as major drivers for migration.^59^^,^^60^ To bridge the gap between acute workforce shortages and access to care, further community-oriented training for oral health professionals is necessary, emphasizing prevention and health promotion within integrated public health services.^61^, ^62^, ^63^, ^64^

The establishment of the NHIF in 2005 aimed to include vulnerable population groups historically reliant on out-of-pocket payments into essential oral health services. However, barriers to enrolment persist due to geographical and organizational constraints. Individual assistance strategies and community outreach initiatives can facilitate enrolment by disseminating information about the NHIF and providing resources to the public.^65^ Additionally, long-term insurance plan subsidies and free preventive care treatments for vulnerable groups, such as children, can alleviate financial burdens.^66^, ^67^, ^68^

This study was conducted during and after one of the waves of the COVID-19 pandemic, at which stage there may have been changes in the health system as lessons are learnt.

To conclude, coordinated interventions can drive oral health policies and planning away from the traditional model of restorative dentistry towards primary prevention and oral health promotion integrated across all levels of the healthcare system, ultimately advancing towards UHC. Improving access entails addressing health inequalities, expanding the role of oral health allied professionals, ensuring financial sustainability through cost containment measures, and facilitating patient enrolment in existing health insurance schemes.

## Conflict of interest

None.

## Appendix Supplementary materials

Image, application 1

## Footnotes

Supplementary material associated with this article can be found, in the online version, at doi:10.1016/j.identj.2024.05.012.
