# Supplementary material for: A Descriptive Review of the Healthcare System and the Provision of Oral Healthcare in the Republic of Sudan
Source: Int Dent J. 2024 Jul 20;74(5):930–6. doi: 10.1016/j.identj.2024.05.012 (PMC11561511; doi:10.1016/j.identj.2024.05.012)
Supplement: Supplementary file 1 [file mmc1.docx]

**Supplementary Material**

eTable 1: Premiums by Sectors. National Health Insurance Fund

| **Sector** | **Payment** | **Source of financing** |
| --- | --- | --- |
| Formal (public-private) | 10% of fixed salary  (4% employee and 6% employer) | MoF* |
| Self-employed | Flat rate for family | Syndicates  Associations  Unions |
| Poor families | Flat rate for families | Zakat^#^  NGOs  MoF |
| Pensioners | Flat rate for families | MoF  NHIF |

*Ministry of Finance; ^#^ Zakat is one of the five pillars of Islam and considered a religious duty for wealthy people to help those in need through financial or in-kind contributions. Muslims earning over SDG10,000 (about USD3,575) per year are required to pay 2.5 per cent of their annual income to the Zakat Fund. Non-Muslims pay a social solidarity tax at an equivalent rate. Covered under the Zakat Act of 2001, Zakat is compulsory in Sudan. The Ministry of Welfare and Social Security manages the Zakat Fund through the Zakat Chamber, which operates as a semi-autonomous agency. The Fund has an extensive institutional infrastructure that reaches all the way down to the village level. Cash and in-kind revenues are collected from all over the country, administered centrally and then redistributed through state and local administrative structures (source: Anna Carolina Machado, Charlotte Bilo and Imane Helmy, 2018. "[The role of zakat in the provision of social protection: a comparison between Jordan, Palestine and Sudan](https://ideas.repec.org/p/ipc/wpaper/168.html)," [Working Papers](https://ideas.repec.org/s/ipc/wpaper.html) 168, International Policy Centre for Inclusive Growth).

eTable 2: Dental Facilities and Personnel Distribution. Federal Ministry of Health, March 2020.

|  | **State** | **Population** | **Dentists** | **Specialists** | **Dental assistant** | **Private clinics** | **PHCs** | **N° of departments** | **Dental Hospitals** |
| --- | --- | --- | --- | --- | --- | --- | --- | --- | --- |
| **1** | **Blue Nile** | 1080743 | 5 | 1 | 6 | 2 | 8 | 6 | - |
| **2** | **White Nile** | 2410260 | 14 | - | 7 | 16 | 14 | 9 | - |
| **3** | **Sennar** | 1847458 | 12 | 1 | 8 | - | 5 | 2 | - |
| **4** | **South Kordofan** | 1458228 | 2 | - | 6 | 14 | 4 | 9 | - |
| **5** | **West Kordofan** | 1197830 | 5 | 1 | 7 | - | 5 | 6 | - |
| **6** | **North Kordofan** | 2551899 | 18 | 3 | 5 | 7 | 34 | 6 | - |
| **7** | **South Darfur** | 4071749 | 7 | 1 | 8 | 10 | 18 | 1 | - |
| **8** | **North Darfur** | 2296068 | 7 | 2 | 17 | 10 | 6 | 18 | - |
| **9** | **West Darfur** | 1000564 | 6 | - | 4 | 5 | 6 | 1 | - |
| **10** | **East Darfur** | 1148442 | 2 | 1 | 2 | - | 4 | 1 | - |
| **11** | **Central Darfur** | 724546 | 2 | - | 2 | 1 | 5 | 1 | - |
| **12** | **Kordofan** | 4926555 | 88 | 7 | 74 | 31 | 24 | 44 | 2 |
| **13** | **Nile** | 1472257 | 8 | 1 | 6 | 12 | 21 | 6 | - |
| **14** | **Northern** | 913533 | 7 | - | 30 | 12 | 6 | 27 | - |
| **15** | **Gedaref** | 2108468 | 9 | 1 | 6 | 16 | 3 | 10 | - |
| **16** | **Kassala** | 2438808 | 8 | 1 | 22 | 9 | 21 | 8 | - |
| **17** | **Khartoum** | 7687547 | 235 | 70 | - | 491 | 164 | 4 | 1 |
| **18** | **Red Sea** | 1447787 | 21 | 2 | 3 | 10 | 10 | 6 | - |
| **Total** | | 40.782.742 | 444 | 88 | 213 | 646 | 358 | 160 | 8 |

eTable 3: Sudan Dental School Distribution. University of Khartoum

|  | **State** | **No. of private schools** | **No. of Governmental school** | **Total No. of Schools** |
| --- | --- | --- | --- | --- |
|  | **Khartoum** | 21 | 5 | 26 |
|  | **North Kordofan** | 0 | 1 | 1 |
|  | **Northern** | 0 | 0 | 0 |
|  | **Kassala** | 0 | 0 | 0 |
|  | **Blue Nile** | 0 | 0 | 0 |
|  | **North Darfur** | 0 | 0 | 0 |
|  | **South Darfur** | 0 | 0 | 0 |
|  | **South Kordofan** | 0 | 0 | 0 |
|  | **Al Gezira** | 1 | 2 | 3 |
|  | **White Nile** | 0 | 0 | 0 |
|  | **River Nile** | 0 | 1 | 1 |
|  | **Red Sea** | 1 | 1 | 2 |
|  | **Gadaref** | 0 | 0 | 0 |
|  | **Sennar** | 0 | 0 | 0 |
|  | **West Darfur** | 0 | 0 | 0 |
|  | **Central Darfur** | 0 | 0 | 0 |
|  | **East Darfur** | 0 | 0 | 0 |
|  | **West Kordofan** | 0 | 0 | 0 |
|  | **Total** | **33** | | |
